# Supplementary material for: Anatomical and Biomechanical Properties of the Junction between Stem and Aerial Roots of Selenicereus undatus
Source: Plants (Basel). 2023 Jul 4;12(13):2544. doi: 10.3390/plants12132544 (PMC10346808; doi:10.3390/plants12132544)
Supplement: Supplementary file 1 [file plants-12-02544-s001.zip › S2.pdf]

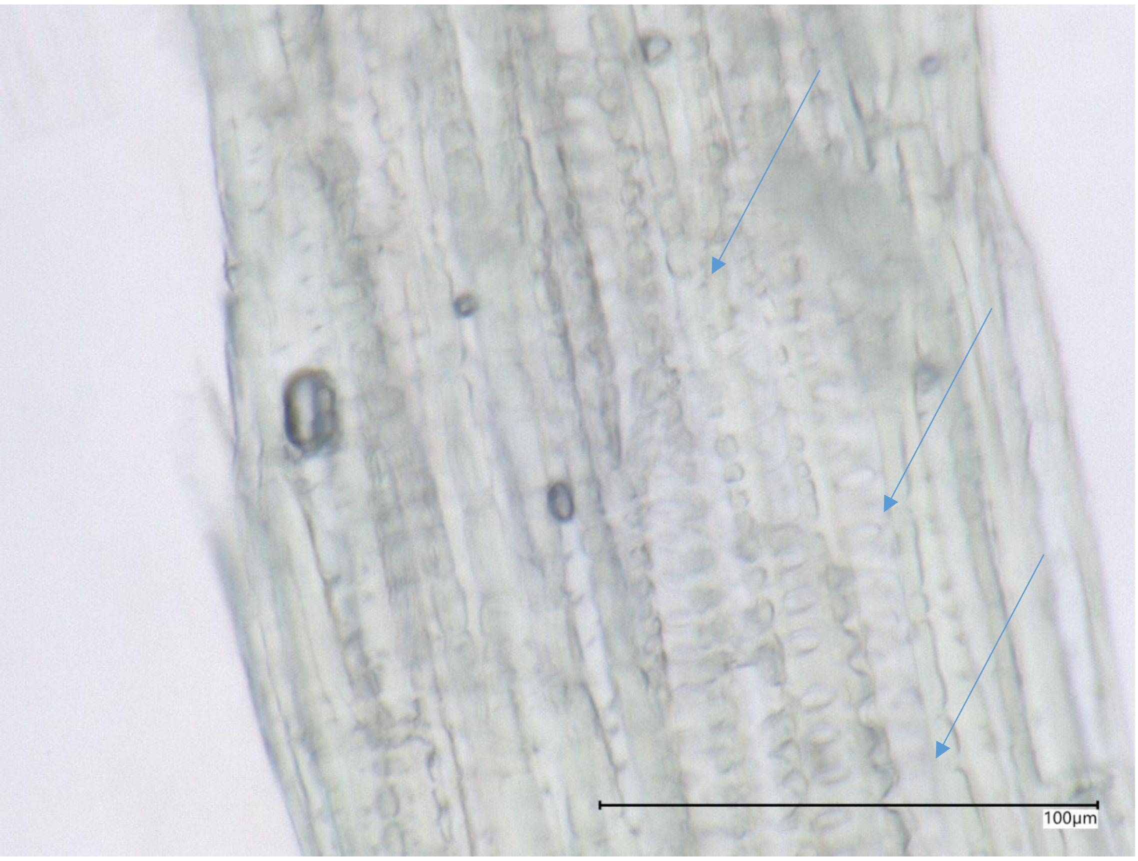


**Supplementary Figure S1:** Longitudinal section of *Selenicereus undatus* aerial root. Perforated vessel elements marked with arrows.

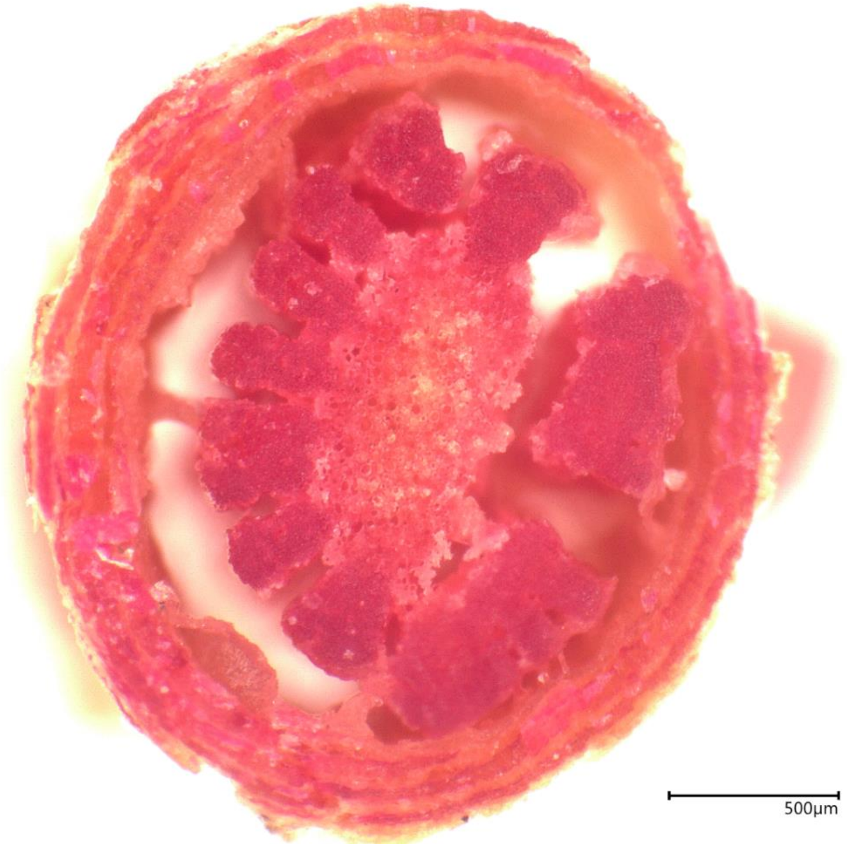


**Supplementary Figure S2:** Result of phloroglucinol HCl staining on mature *Selenicereus undatus* aerial root cross section. Presence of lignin in all root parts is confirmed by the red color.

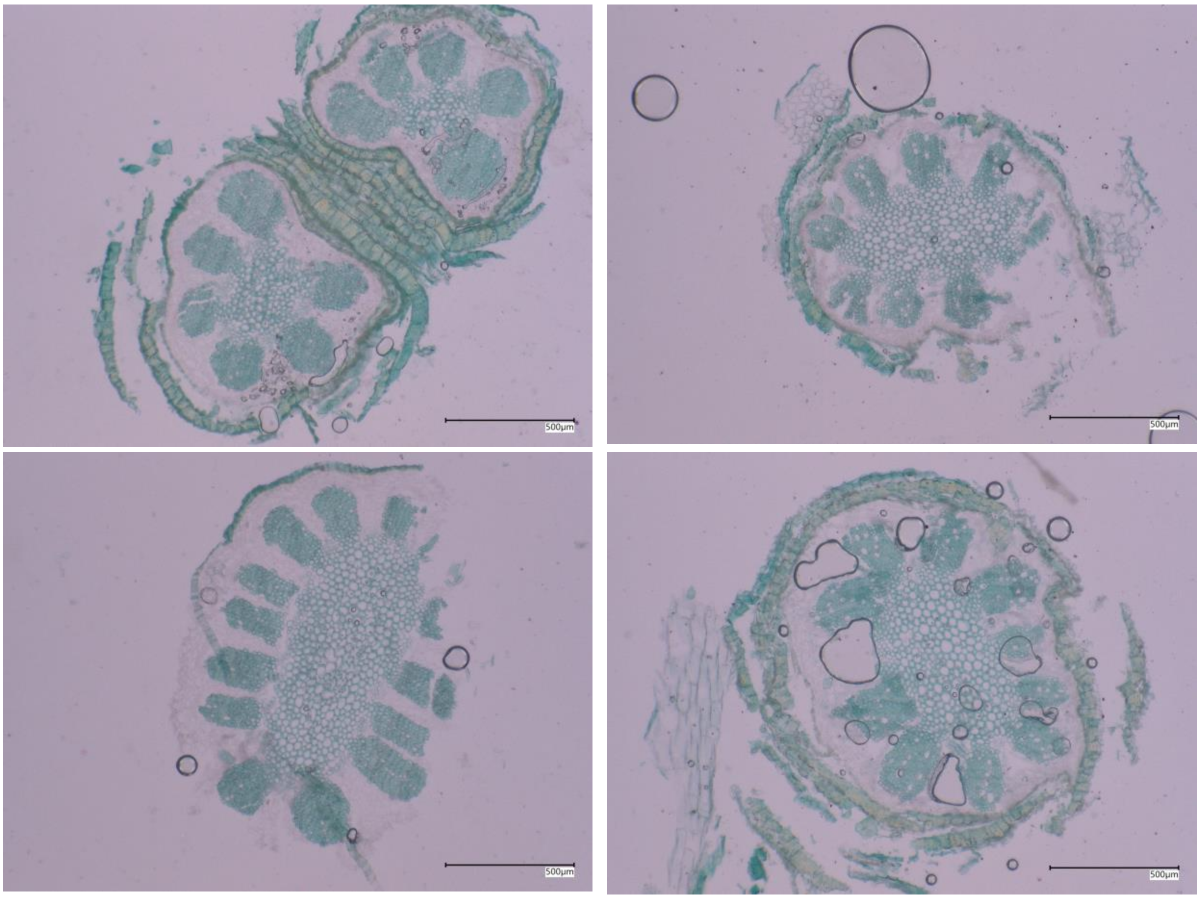


**Supplementary Figure S3:** Cross sections of different aerial roots of *Selenicereus undatus*. The partially missing bast sheath in some sections is a consequence of the sectioning process.

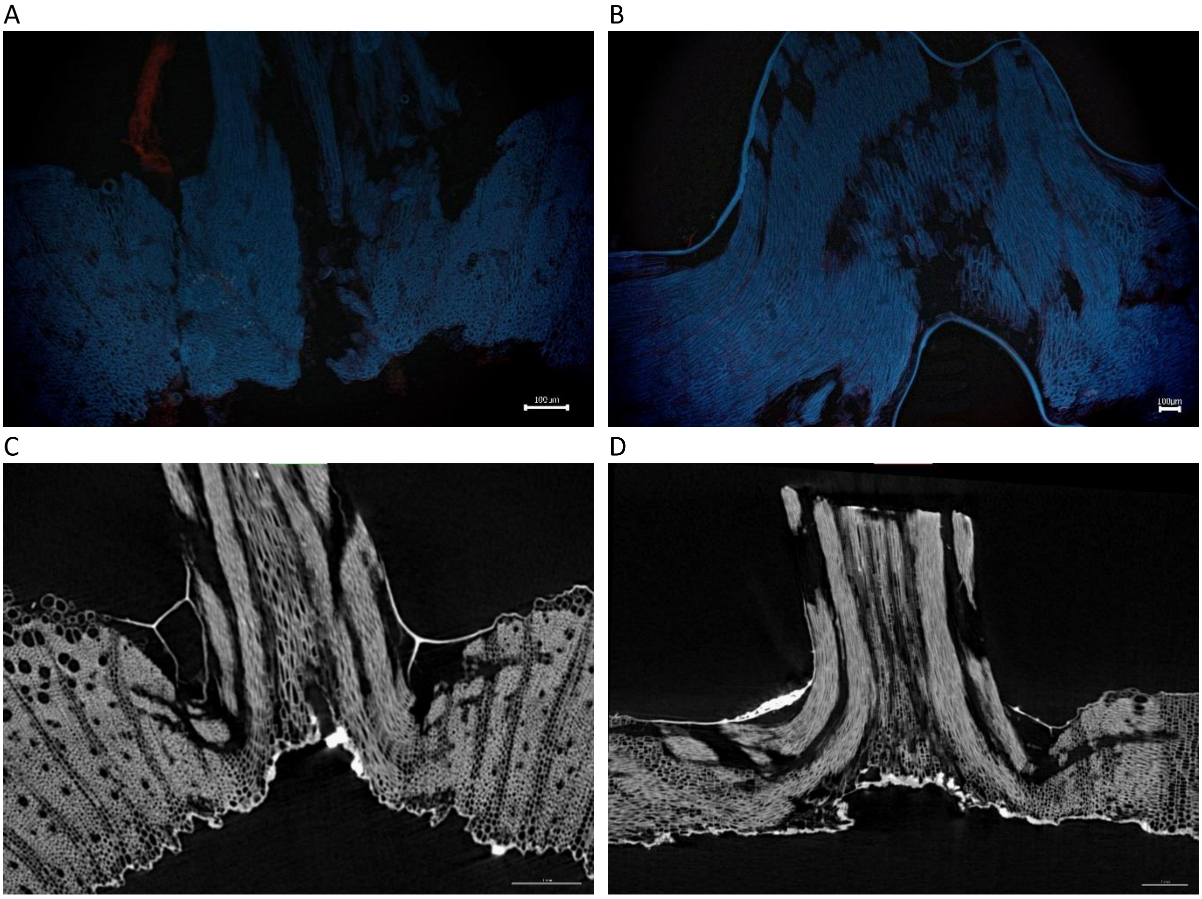


**Supplementary Figure S4:** Comparison of microscopic sections (A and B) and comparable sections extracted from the X-ray imaging (C and D) at the point of the aerial root junction. A and C cross sections of the central wooden cylinder, B and D longitudinal sections. Microscopic sections stained with carmine-methylgreen and imaged using UV-fluorescence as described in [31].
